# National Birth Defects Prevention Month and Folic Acid Awareness Week — January 2015

**Published:** 2015-01-16

Birth defects affect about one in 33 newborns in the United States (1). This year, National Birth Defects Prevention Month focuses on “Making Healthy Choices to Prevent Birth Defects — Make a PACT for Prevention: Plan ahead, Avoid harmful substances, Choose a healthy lifestyle, and Talk to your doctor.”

Health care providers should encourage women to plan for pregnancy; avoid harmful substances, like tobacco (2) and alcohol (3); and choose a healthy lifestyle, like eating a healthy diet (4), to increase their chances of a healthy pregnancy. Health care providers should also discuss with women any medications they might be taking, both prescription and over-the-counter, to ensure they are taking only what is necessary. More information is available at http://www.cdc.gov/ncbddd/birthdefects/prevention.html.

January 4–10, 2015, is National Folic Acid Awareness Week. CDC urges all women of childbearing age who can become pregnant to get 400 *μ*g of folic acid every day to help reduce the risk for neural tube defects (major birth defects of the brain and spine). Health care providers should encourage women to consume folic acid in fortified foods or supplements, or a combination of the two, in addition to a diet rich in folate. More information about folic acid is available at http://www.cdc.gov/folicacid.
